# Complex Toxin Profile of French Mediterranean *Ostreopsis* cf. *ovata* Strains, Seafood Accumulation and Ovatoxins Prepurification

**DOI:** 10.3390/md12052851

**Published:** 2014-05-13

**Authors:** Charline Brissard, Christine Herrenknecht, Véronique Séchet, Fabienne Hervé, Francesco Pisapia, Jocelyn Harcouet, Rodolphe Lémée, Nicolas Chomérat, Philipp Hess, Zouher Amzil

**Affiliations:** 1 Ifremer, Phycotoxins Laboratory, rue de l’Ile d’Yeu, BP 21105, Nantes F-44311, France; E-Mails: veronique.sechet@ifremer.fr (V.S.); Fabienne.Herve@ifremer.fr (F.H.); francescopisapia90@gmail.com (F.P.); jocelyn.harcouet@free.fr (J.H.); Philipp.Hess@ifremer.fr (P.H.); zouher.amzil@ifremer.fr (Z.A.); 2 L’Université Nantes Angers Le Mans (LUNAM), University of Nantes, MMS EA2160, Pharmacy Faculty, 9 rue Bias, Nantes F-44035, France; E-Mail: christine.herrenknecht@univ-nantes.fr; 3 Sorbonne Universités, UPMC Univ Paris 06, UMR 7093, LOV, Observatoire Océanologique, Villefranche/mer F-06230, France; E-Mail: lemee@obs-vlfr.fr; 4 CNRS, UMR 7093, LOV, Groupe Biodiversité et Biogéochimie, Observatoire océanologique, Villefranche/mer F-06230, France; 5 Ifremer, Laboratoire Environnement Ressource de Bretagne Occitentale (LER-BO), Marine Biological Station, BP 40537, Concarneau F-29185, France; E-Mail: Nicolas.Chomerat@ifremer.fr

**Keywords:** *Ostreopsis* cf. *ovata*, palytoxin & ovatoxins, culture, seafood contamination, LC-MS/MS, hemolysis assay, Mediterranean Sea

## Abstract

*Ostreopsis* cf. *ovata* produces palytoxin analogues including ovatoxins (OVTXs) and a putative palytoxin (p-PLTX), which can accumulate in marine organisms and may possibly lead to food intoxication. However, purified ovatoxins are not widely available and their toxicities are still unknown. The aim of this study was to improve understanding of the ecophysiology of *Ostreopsis* cf. *ovata* and its toxin production as well as to optimize the purification process for ovatoxin. During *Ostreopsis* blooms in 2011 and 2012 in Villefranche-sur-Mer (France, NW Mediterranean Sea), microalgae epiphytic cells and marine organisms were collected and analyzed both by LC-MS/MS and hemolysis assay. Results obtained with these two methods were comparable, suggesting ovatoxins have hemolytic properties. An average of 223 μg·kg^−1^ of palytoxin equivalent of whole flesh was found, thus exceeding the threshold of 30 μg·kg^−1^ in shellfish recommended by the European Food Safety Authority (EFSA). *Ostreopsis* cells showed the same toxin profile both *in situ* and in laboratory culture, with ovatoxin-a (OVTX-a) being the most abundant analogue (~50%), followed by OVTX-b (~15%), p-PLTX (12%), OVTX-d (8%), OVTX-c (5%) and OVTX-e (4%). *Ostreopsis* cf. *ovata* produced up to 2 g of biomass per L of culture, with a maximum concentration of 300 pg PLTX equivalent cell^−1^. Thus, an approximate amount of 10 mg of PLTX-group toxins may be produced with 10 L of this strain. Toxin extracts obtained from collected biomass were purified using different techniques such as liquid-liquid partition or size exclusion. Among these methods, open-column chromatography with Sephadex LH20 phase yielded the best results with a cleanup efficiency of 93% and recovery of about 85%, representing an increase of toxin percentage by 13 fold. Hence, this purification step should be incorporated into future isolation exercises.

## 1. Introduction

Palytoxin (PLTX) is one of the most potent marine toxins known so far with remarkable biological activity at an extremely low concentration [1]. Palytoxin group toxins (PLTX-group toxins) were first reported in Hawaii and Japan, but are currently known to be distributed worldwide [2,3]. PLTX was first isolated from the soft coral *Palythoa toxica*, and was subsequently found in several tropical *Palythoa* species [1,4,5]. Since that discovery, many other organisms have been identified as producers of PLTX analogues, including those belonging to dinoflagellate of the genus *Ostreopsis* [6], together with some analogues [7,8,9,10]. Originally found in tropical areas, these dinoflagellates have been shown to proliferate since the early 2000s around the Mediterranean basin with increasing frequency, intensity and distribution [11,12,13,14]. The presence of *Ostreopsis* spp. in temperate areas is still unclear. While the first report of *Ostreopsis* sp. in the Mediterranean dates back to 1972 [15], it is debatable whether the increased occurrence of high density blooms can be attributed to climate change or whether other factors need to be considered as well [16].

Species of the genus *Ostreopsis* are benthic dinoflagellates living in close contact with a variety of biotic or abiotic substrata such as microalgae, sea-grasses, benthic invertebrates, sand and rocks [17]. Cells produce abundant mucilaginous matrix and are often embedded inside this mucus [18], and both free cells and mucus can also be found in the water column. High temperature and low turbulence influence proliferation of *Ostreopsis* spp. which grows better in shallow water [19,20]. *Ostreopsis* spp. can bloom and form floating clusters at the surface and provoke respiratory problems or irritation for bathers, by inhalation or cutaneous contact respectively. Moreover, occurrence of *Ostreopsis* spp. may result in contamination of marine organisms intended for human consumption [21]. *Ostreopsis* toxins can enter the food web and accumulate in several groups of organisms including crustaceans, mollusks, fishes and echinoderms and can lead to food intoxication for consumers [22,23,24,25]. The first events in Europe occurred in 2005 in Italy, when hundreds of people required medical attention after exposure to marine aerosol coinciding with intense proliferation of *Ostreopsis* cf. *ovate* (1.6 × 10^6^ cell·L^−1^) [26]. In the French Mediterranean coast, *Ostreopsis* cf. *ovata* has regularly proliferated since 2006. The first sanitary report in France involving *Ostreopsis* cf. *ovata* dated back to August 2006, when four divers suffered from mouth and throat irritation symptoms with fever after diving in Frioul Island in the Marseille offshore area [27]. Simultaneously, *Ostreopsis* cf. *ovata* cells were found in the water column, with cell concentrations around 3.8 × 10^4^ cell·L^−1^. Since that time, specific monitoring was designed along the French Mediterranean coast and a concentration limit of 4000 cell·L^−1^ in the water column now triggers PLTX-group toxins chemical analysis by LC-MS/MS in shellfish. However, there are no regulations on PLTX-group toxins in shellfish, either in the European Union (EU), or in other regions of the world. In a first risk assessment, the European Food Safety Authority (EFSA) recommended a threshold of 30 μg/kg, based on a seafood portion of 400 g [28].

**Figure 1:**
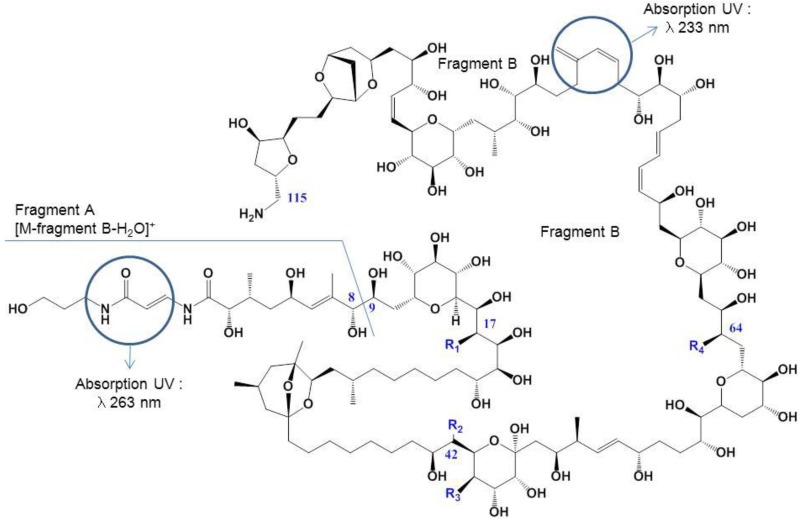
Palytoxin (PLTX) and ovatoxin (OVTX)-a structures.

*Ostreopsis* cf. *ovata* is a producer of PLTX-group of toxins including ovatoxins (OVTXs) namely ovatoxin-a, -b, -c, -d-, -e, -f and a putative-palytoxin (p-PLTX) [9,26,29]. PLTX-group toxins are large and complex polyhydroxylated compounds with both hydrophilic and lipophilic parts. The molecule consists of a long partially unsaturated aliphatic backbone containing 10 cyclic ethers, 64 chiral centers, 40–42 hydroxyl and two amide groups (Figure 1). However, the primary amino-group at the C115 end of the molecule accounts for the basicity of PLTX-group toxins [30]. Molecular weights of PLTXs range from 2659 to 2680 Daltons (Da) depending on the origin [31]. PLTX-group toxins exhibit ultraviolet absorption spectrum with λ_max_ at 233 and 263 nm due to their two chromophores (Figure 1). Among ovatoxins, only the structure of ovatoxin-a (Figure 1) was elucidated by high resolution mass spectroscopy (HR-MS*^n^*) [29] and nuclear magnetic resonance (NMR) [32]. For the others ovatoxins, only structural differences with ovatoxin-a are known (Table 1). The purification of ovatoxin standards should be undertaken to provide reference material for method development, routine monitoring and toxicological assessment.

**Table 1:** Structural differences between palytoxin (PLTX) and ovatoxins (OVTXs) [9,29].

Toxins	Elementary Formulae	Differences with PLTX	MW Da
Palytoxin	C_129_H_223_N_3_O_54_		2678.48
Ovatoxin-a	C_129_H_223_N_3_O_52_	−2 O	2646.49
Ovatoxin-b	C_131_H_227_N_3_O_53_	+C_2_H_4_; −1 O	2690.52
Ovatoxin-c	C_131_H_227_N_3_O_54_	+C_2_H_4_	2706.51
Ovatoxin-d/-e	C_129_H_223_N_3_O_53_	−1 O	2662.49
Ovatoxin-f	C_131_H_227_N_3_O_52_	+C_2_H_4_; −2 O	2674.52

Several published techniques are used for the determination of PLTX-group toxins in marine organisms [33]. The mouse bioassay (MBA) is considered a simple method for detection of PLTX-group toxins, but for ethical and scientific reasons, other biological methods have been developed [28]. A specific biological property of PLTX is its ability to interact with the Na^+^/K^+^-ATPase of mammalian cells converting these ion-specific pumps into non-selective cationic pores [34,35]. This interaction leads to ion imbalance in red blood cells resulting in delayed hemolysis [36]. This property is used for the hemolysis assay with erythrocytes, following hemoglobin release into the extracellular media. The hemolysis induced by palytoxin is specifically inhibited by ouabain in most cell types, a glycoside able to bind to the Na^+^/K^+^ pump instead of palytoxin [37] allowing the matrix effect to be measured. In addition to biological methods, analytical techniques have been used, in particular liquid chromatography coupled with a mass spectrometer (LC-MS) [10,12,26]. Due to lack of calibration standards for ovatoxins, LC-MS results are typically expressed as palytoxin equivalent, assuming that molecules of PLTX-group toxins possess the same molecular response [26], although ovatoxins represent 90% of the toxin profile produced by *Ostreopsis* cf. *ovata*. As this practice relies on the assumption that all analogues have the same response factor in MS detection, ovatoxins need to be purified and isolated.

The aim of this work was first to complete the investigation of the *Ostreopsis* cf. *ovata* profile in natural cell samples from the French Mediterranean coast and to determine PLTX-group toxin levels in marine organisms, as well as to determine indirectly the hemolytic power of total OVTXs. After isolation of *Ostreopsis* cells collected in the Villefranche-sur-Mer bay and optimization of toxic cultures, pellets of *Ostreopsis* cells were obtained. The biomass was used to develop a purification protocol.

## 2. Results and Discussion

### 2.1. Ostreopsis cf. ovata Blooms and Accumulation in Marine Organisms

Since 2006, results of French monitoring have revealed that *Ostreopsis* cf. *ovata* regularly proliferates every year in summer, as also reported by several authors [25,38,39,40]. To evaluate toxin profiles *in situ*, *Ostreopsis* cf. *ovata* cells and marine organisms were harvested in summer 2011 and 2012. 

#### 2.1.1. Recurrence of *Ostreopsis* cf. *ovata* Bloom on the French Mediterranean Coast

Several sampling campaigns have been carried out on a site with a rocky substrate (Villefranche-sur-Mer) where *Ostreopsis* cf. *ovata* had been previously shown to bloom regularly [25,39,40,41]. *Ostreopsis* cf. *ovata* cells were sampled by snorkeling every week from the beginning of July to the end of August, both in the water column (at about 0.3 m deep) and on macroalgae (at about 0.5 m deep) and then counted.

**Figure 2:**
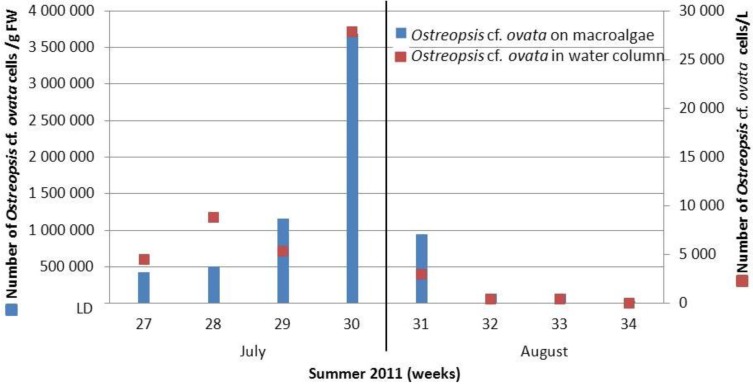
*Ostreopsis* cf. *ovata* cells concentration in the water column (cells·L^−1^) and on macroalgae *Stypaucolon scoparium* (cells·g^−1^·FW) in summer 2011 at Villefranche sur Mer bay. Average of three points.

*Ostreopsis* cf. *ovata* blooms appeared in the last week of July (Figure 2), in accordance with the main blooming period reported in the North-West Mediterranean Sea [40,41]. Year 2011 was characterized by an important bloom, with tenfold higher epiphytic cell numbers than during the bloom of 2012. Indeed, at the end of July 2011, the maximum cell abundance reached 3.7 × 10^6^ cells·g^−1^ fresh weight (FW) and 28 × 10^3^ cell·L^−1^ for epiphytic and planktonic cells respectively, whereas cell concentrations reached 4.9 × 10^5^ cells·g^−1^·FW for epiphytic cells on the last week of July 2012 and 7× 10^3^ cell·L^−1^ for planktonic cells in early August 2012.

**Figure 3:**
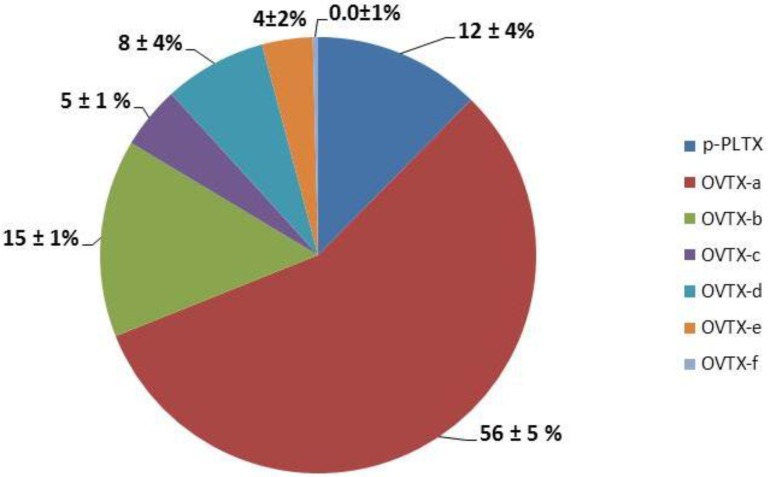
PLTX-group toxins profile of *Ostreopsis* cf. *ovata* sampled at 0.5 m depth during summers 2011 and 2012.

When cell concentrations were maximal, an epiphytic cell collection was carried out in order to evaluate toxin profile and content by LC-MS/MS according to Ciminiello *et al.* [9]. Toxin profiles of samples collected at 0.5 m depth in 2011 and 2012 remained identical (Figure 3) and were dominated by ovatoxin-a as a major component, with an average contribution of 56%; followed by ovatoxin-b (15%), putative palytoxin (12%), ovatoxin-d (8%), ovatoxin-c (5%), ovatoxin-e (4%), ovatoxin-f [29] (<1%). The toxin profile is not similar to the other *Ostreopsis* cf. *ovata* strains reported so far. Indeed, the relative percentage of putative palytoxin is usually 0.1%–3% (here it is 10%) and ovatoxin-f (in the only strain that contained it) accounted for more than 50% of the total content (here it is <1%).

Total toxin content reached 22.5 and 32.8 pg·cell^−1^ in 2011 and 2012, respectively, at 0.5 m depth. These concentrations were in the same order of magnitude as those reported from the Ligurian [42] and Tyrrhenian Seas [43,44], but lower than those found in the northern Adriatic Sea (72–75 pg·cell^−1^) [45].

#### 2.1.2. Influence of Depth on Toxin and Cell Concentration in the Field

*Ostreopsis* cf. *ovata* blooms represent a potential risk in recreational waters due to the presence of toxic cells. A question to be put is how depth influences cell growth and toxin production. As already reported, *Ostreopsis* proliferation decreases with depth, with maximum abundance between the surface and 5 m depth [46]. Therefore, the toxin profile and concentration were investigated in the upper meters of depth in recreational areas.

**Figure 4:**
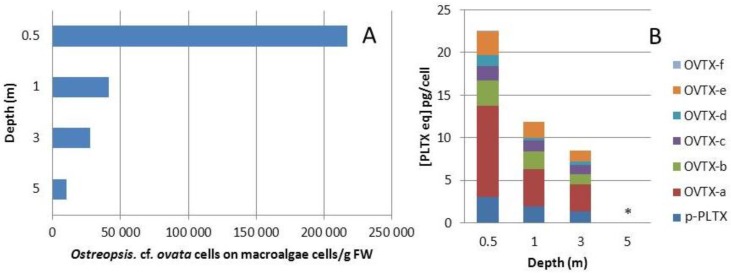
*Ostreopsis* cf. *ovata* sampled week 30 in 2011: (**A**) *Ostreopsis* proliferation between the surface and 5 m depth on brown macroalgae *Padina pavonica*; and (**B**) Toxin concentration and profile of *Ostreopsis* cf. *ovata* between the surface and 3 m depth (same sample, on *P. pavonica*) * at 5 m depth, cell number was too low for toxin quantification.

In 2011, *Ostreopsis* cf. *ovata* was harvested during the second half of July (week 30). Cell concentrations on macroalgae (*Padina pavonica*) were measured at 0.5, 1, 3 and 5 m depth (Figure 4A). Toxin contents were also evaluated up to 3 m depth (Figure 4B). Indeed, at 5 m depth, toxin concentration was below the detection limit, because of the small number of cells; therefore the toxin profile could not be reported. Moreover, pelagic cells were low in abundance in water samples; with toxin concentrations below the limit of detection.

Cell concentrations decreased with increasing depth. At 0.5 m depth, cell concentration reached 2.17 × 10^5^ cell·g^−1^·FW, and was reduced fivefold to 4.13 × 10^4^ cell·g^−1^·FW at 1 m depth. Cell concentration at 3 m deep was in the same order of magnitude of 1 m deep cell concentration, around 2.74 × 10^4^ cell·g^−1^·FW; and finally decreased to 9.92 × 10^3^ cell·g^−1^·FW at 5 m deep. A computed correlation (Sigmaplot 12.5) fits an inverse second order polynomial (*r*^2^ = 0.9923), see Supplementary Information. This *Ostreopsis* depth distribution showed the same pattern as found in the literature, with higher development from the surface to 2 m, followed by significant decrease of abundances from 3 m [14,46]. From 0.5–1 to 3 m deep, total toxin content per cells regularly decreased from 22.5 to 11.9, and finally to 8.5 pg·cell^−1^ respectively. Thus, cells produced less toxins with increasing depth, a finding that corroborates previous studies in which high irradiance influence positively correlated with toxin production, both in cultured and field samples [27,46,47]. The toxin profile of collected samples (in triplicate) remained approximately the same, independent of depth. The samples were dominated by ovatoxin-a as a major component (37%–48%); followed by putative-palytoxin (15%–13%), ovatoxin-b (13%–17%), ovatoxin-e (12%–16%), ovatoxin-c (8%–12%), ovatoxin-d (2%–5%) and ovatoxin-f (0%–0.2%). The slight variation of toxin profile suggests that toxin metabolism is not affected by depth, contrary to toxin content. To our knowledge, this is the first report of toxin profile and concentration as a function of depth for *Ostreopsis* cf. *ovata*.

#### 2.1.3. Accumulation of PLTX-Group in Marine Organisms

In 2012, to evaluate toxin accumulation levels during *Ostreopsis* blooms, a wide variety of faunal species were sampled at Villefranche-sur-Mer, extracted and analyzed by liquid chromatography coupled with tandem mass spectrometry (LC-MS/MS). When *Ostreopsis* cf. *ovata* reached 2.7 × 10^5^ cells·g^−1^·FW, gastropods, echinoderms and fishes were caught by snorkeling or fishing from the shore: 10 red mouthed rock shells (*Stramonita haemastoma*), 10 sea urchins (*Paracentrotus lividus*), 21 sea-breams (*Sarpa salpa*) and four stripped red mullets (*Mullus surmuletus*) were sampled. For sea urchins and fishes, digestive tube (DT) and remaining tissue (RT) were analyzed separately and results were expressed as whole flesh (WF).

Neither striped red mullets nor red-mouthed rock shells contained detectable amounts of putative-palytoxin or analogues. Toxin accumulation was mainly found in sea urchins and sea breams with the ratio of contaminated over total number of organisms being 9/10 and 6/21 for sea urchins and sea-breams respectively (see Supplementary Information for contamination levels and organism sizes). This difference in contamination between urchins and fish can most probably be explained by the fact that the former are grazers [48]. Indeed, sea-urchins and sea-breams are herbivorous, they graze directly on macroalgae on which *Ostreopsis* cf. *ovata* proliferate, whereas red mouthed rock shells and stripped red mullets are carnivorous.

Ovatoxins were detected only in DT. Sea urchin and sea bream RTs were not found contaminated as previously reported [25,48]. Levels in contaminated sea urchins ranged from 103 to 423 μg·kg^−1^ of whole flesh, with an average of 223 μg·kg^−1^ that revealed high variability of contamination levels between individual organisms. Hence, in a monitoring network, at least 10 individuals should be collected to have a good overview of sea urchin toxin contamination levels [48]. For contaminated sea breams, the contamination levels were between 33 and 152 μg·kg^−1^ in the whole flesh. These contamination levels were similar to those already described in sea urchins and determined by LC-MS [24,25], and also similar to those described in sea bream and determined by hemolytic assay [48].

Some samples were also analyzed by hemolytic assay to allow for direct comparison with LC-MS. The hemolytic assay is based on the detection of hemoglobin released from sheep erythrocytes, following exposure to hemolytic compounds. Samples were analyzed both with erythrocytes pre-incubated with ouabain, a known palytoxin antagonist, to distinguish between the matrix effect and without ouabain, to determine the non-specific hemolysis. Marine organism extracts were first diluted 40 fold to avoid matrix effects.

Among contaminated sea urchins and sea breams, some extracts, both DT and RT, were analyzed using the hemolytic test. Results were expressed as whole flesh (WF) (Table 2). In accordance with LC-MS/MS analyses, no RTs were hemolytic. During the experiment with sea breams DT, quantitation of PLTX-group toxins in these samples was impossible due to the high matrix effect. However, amounts of PLTX-group toxins in sea urchin DTs were similar to those found by LC-MS/MS.

**Table 2:** Quantification comparison of sea urchins digestive tube (DT) concentration (WF) analyzed by LC-MS/MS and by hemolytic test in μg of PLTX equivalent per kg of whole sea urchin.

**Whole Flesh Sea Urchin** *Paracentrotus Lividus*	**By LC-MS/MS μg·kg**^−1^	**By Hemolytic Test μg·kg**^−1^
	231	247
	309	270
	205	201
	215	179

Knowing that ovatoxins represent typically 90% of the total toxin profile, these results suggest that ovatoxins are as hemolytic as palytoxin as already reported by Pezzolesi *et al.* [49]. As PLTX targets membrane sodium-potassium pumps (Na^+^/K^+^-ATPase), responsible for maintaining ion gradients critical to cellular function, and as this pump is present in numerous cells, such as nervous cardiac and muscular cells, it is important to confirm the toxicological effect of OVTXs.

Due to the lack of pure ovatoxins, the toxicity of each toxin individually is not known. Purification of toxins is therefore necessary. Such purification should be easier from *Ostreopsis* cells rather than from contaminated animal tissues. For this reason, these cells were isolated and cultivated in the laboratory to obtain the toxins.

### 2.2. Ostreopsis cf. ovata Culture

#### 2.2.1. Genetic Characterization of *Ostreopsis* Strains

**Figure 5:**
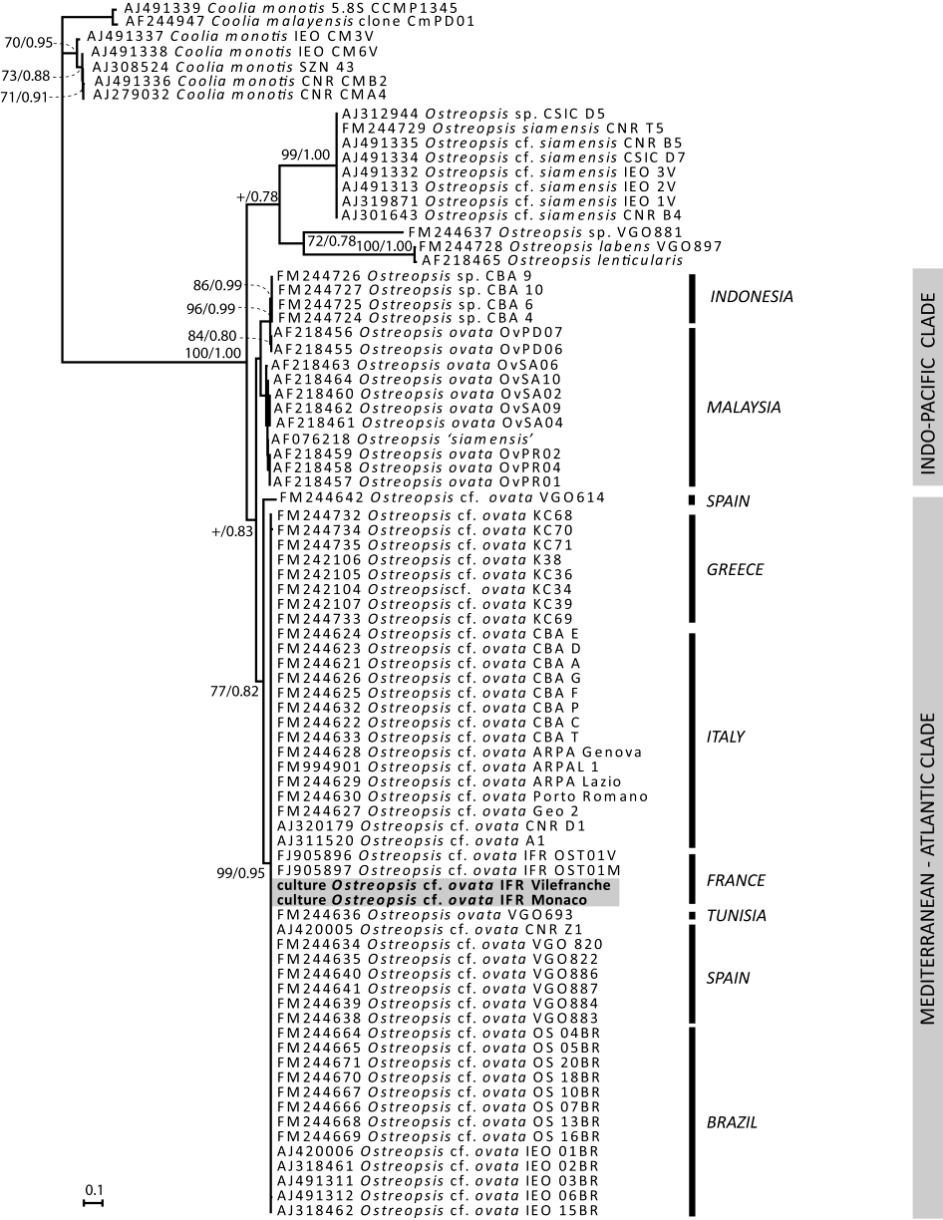
Phylogenetic tree of French *Ostreopsis* cf. *ovata*.

*Ostreopsis* strains cultivated in the laboratory were identified by genetic molecular analysis of the ITS1-5.8S-ITS2 region and partial LSU (D1–D3), which had already been used for genetic identification of *Ostreopsis* species [50]. Sequences obtained for the strains from Monaco IFR-OST01MO (Genbank #KJ239219) and from Villefranche-sur-Mer IFR-OST03V (Genbank #KJ239220) were 1272 bp long and 100% identical. In the phylogenetic tree inferred from ITS1-5.8S-ITS2 (Figure 5), the sequences clustered with the sequences of other *Ostreopsis* cf. *ovata* from France and from various areas of the Mediterranean Sea, especially Italy and Spain, and the Atlantic Ocean. It is genetically different from the Asian clade and since no sequence from the type locality is available, it is not possible to ascertain which genotype actually corresponds to *Ostreopsis* cf. *ovata* and hence our strain is referred to as *Ostreopsis* cf. *ovata*.

#### 2.2.2. Cell Densities in Sea Water and Toxin Profiles of *Ostreopsis* cf. *ovata* Strains

Batch cultures of *Ostreopsis* cf. *ovata* strains from different localities of the French Mediterranean Sea had previously been established to follow algal growth and toxin production and also to optimize culture conditions [27]. This study highlighted the high level of irradiance needed for optimal photosynthetic activity; therefore, an irradiance of 420 μmol·m^−2^·s^−1^ was selected for algal cultures. These authors also demonstrated that growth was enhanced using L1 medium with soil extract, which also promoted the toxin production in the cultures of *Ostreopsis* cf. *ovata.* At the time of these studies, only OVTX-a and p-PLTX were analyzed in the *Ostreopsis* extract. Therefore, French strains from Morgiret, Villefranche and Monaco were cultivated with the optimized culture conditions. Every 25–30 days, when cultures had reached the stationary phase, cultures were harvested, cells were counted and the contents of PLTX-group toxins were determined.

At the stationary phase, cell densities varied between 10,500 and 18,800 cell·mL^−1^ and toxin concentrations varied between 75 and 143 pg·cell^−1^, with a maximum concentration reaching 300 pg·cell^−1^ in the case of the Villefranche strain. Toxin profiles were also evaluated, ovatoxin-a being by far the major component of the toxin profile (about 55% of the total toxin content for Villefranche strain and about 71% for Morgiret strain). p-PLTX and OVTX-b, -c, -d, -e were also found, their total percentage varying between 45% and 25%. In our cultures, ovatoxin-f was produced in very low quantity, *i.e.*, less than 1%. The toxin profiles of the Villefranche strain were approximately the same both in culture and in field samples. These values compared well with those previously reported; e.g., Pistocchi *et al.* (2011) indicated cell concentrations of around 13,000–16,000 cell·mL^−1^ in the stationary phase [19]. However, toxin contents in our study were higher than those described in the literature (20–130 pg·cell^−1^ for Tyrrhenian and Adriatic strains [51]).

It is noteworthy that, with the OST-IFR-0.3V strain and our optimized culture conditions, PLTX-group toxins represented about 0.5% (m/m) of the wet extracted mass, after removal of culture media containing salts. A culture of 10 L yielded about 20 g of cell biomass, contaminated with about 10 mg of toxins. This quantity is sufficient to envisage preparative purification.

#### 2.2.3. Toxin Content as a Function of Growth Stage

**Figure 6:**
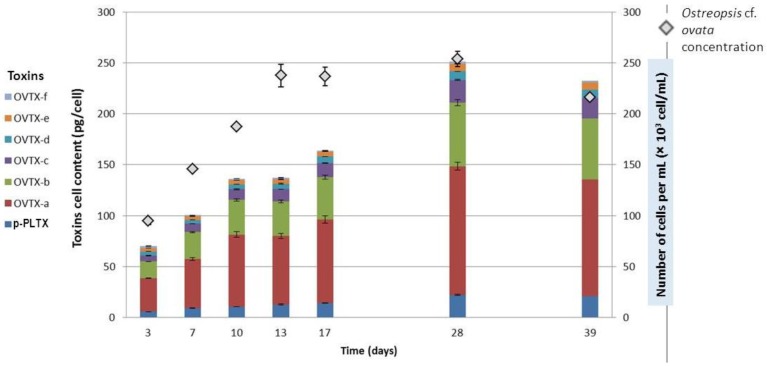
Cell concentration, toxin profile and toxin concentration during the growth curve of OST-IFR-0.3V strain (three replicate aliquots were taken from each culture flask; error bars represent standard deviations).

The amount of toxin present in a culture depends both on toxin content per cell and cell number. The growth of *Ostreopsis* cf. *ovata* strains was analyzed every 3–4 days from the beginning of the exponential phase to the end of the stationary phase. Cell density and toxin content were investigated. As the evaluation of growth of a benthic species is difficult, a specific design, inspired by Guerrini et al. [43], was carried out. A number of 14 *Ostreopsis* culture flasks were grown in parallel. Every other day, two flasks were sampled entirely, one to evaluate cell concentration in triplicate, the other one to determine the toxin content, also in triplicate (Figure 6).

The evolution of cell concentration could be divided into three phases: (i) a linear phase from day 0 to 13, with an average growth rate of 0.26 day^−1^; (ii) then a stationary phase, over about 15 days with cell density around 25,000 cells·mL^−1^; and (iii) a last time point at 39 days that indicated the beginning of a senescence phase. Toxin contents per cell increased during the linear and the stationary phase from 70 to 251 pg·cell^−1^. After 28 days, a slight decrease in toxin content of cells was then observed, probably due to an increased release of toxins in the medium, as already reported in the literature [43]. Toxin profile remained constant, *i.e.*, approximately 50% of ovatoxin-a, 25% ovatoxin-b, 9% of ovatoxin-c, 8% of putative-palytoxin, 4% of ovatoxin-d, 3% ovatoxin-e and 1% of ovatoxin-f.

Previous reports on maximum toxin contents in culture ranged from 140 pg·cell^−1^ for Mediterranean strains [51] to 400 pg·cell^−1^ for a Brazilian strain [52]. It must be noted that, except for the Brazilian study, cultured in 10 mL volumes, we obtained the highest toxin concentration in culture among those using Mediterranean strains. Our culture conditions seemed to be well optimized, favoring both biomass and toxin production per cell. This study led us to conclude that the cells should be harvested after 25–30 culture days.

### 2.3. Purification of Ovatoxins

Ovatoxin-a had been previously isolated by Ciminiello *et al.* (2012), but from a strain producing almost only ovatoxin-a [32]. The *Ostreopsis* strain from Villefranche-sur-Mer produces several ovatoxins, principally OVTX-a, -b, -c and p-PLTX. As ovatoxins possess very close chemical and physical properties, purification of these toxins into separate fractions of individual toxins is difficult. During all the purification steps, quantification using LC-MS/MS and full scan analysis by LC-MS were carried out.

#### 2.3.1. Preparation of *Ostreopsis* cf. *ovata* Extract

Initial trials investigated whether significant portions of toxin were released into the culture medium at different growth stages. Some authors reported a release of toxins into culture media up to 30% at the end of growth [32]. Such proportion should require an extraction method from culture media in order to collect a maximum of toxins. The amphiphilic character of ovatoxins complicated their extraction from culture media. Two protocols were used and compared: (i) liquid-liquid extraction with three times equal volume of butanol and (ii) adsorption and elution through HP20 packed solid phase extraction (SPE) column. The first technique has been commonly used for PLTX-group toxin extraction from media in the literature [26,43]; the second one was previously used in our laboratory to recover other toxins (azaspiracids) from the culture media [53]. Moreover, HP20 resins are described to be suitable for large molecules because of their relatively large pore size. 

Preliminary experiments using seawater spiked with PLTX standard at known concentrations showed a recovery of 60% and 50% for liquid-liquid extraction and adsorption through an HP20 column, respectively (data not shown). As the objective was to have an order of magnitude of the toxin released into the media, these recoveries were considered to be acceptable.

*Ostreopsis* cf. *ovata* cultures were harvested after *ca.* 25 days. Pellets were gently separated from media with scraper to avoid any cell rupture. The culture media was extracted with both methods, the results being corrected with the obtained recovery percentage. Toxin released was less than 5% of total toxin content of the culture, independent of the extraction method. This percentage was considered sufficiently low to consider that toxin extraction from media was not necessary for preparative isolation purposes. Toxins were therefore only extracted from cells. As above-mentioned, harvest after 25–30 days of culture was sufficient to have both high toxin production and cell biomass, associated with a minimum toxin release into the media.

#### 2.3.2. Pre-Purification of *Ostreopsis* cf. *ovata* Extract

After separation from media, cells were broken by sonication, and cellular content was dissolved using aqueous methanol (MeOH/ H_2_O 1:1 v/v). This operation led to a complex extract composed of compounds possessing very low molecular weight (salts and nutrients), very high molecular weight (proteins and polysaccharides) and molecules of intermediate molecular weight. These molecules could have different physico-chemical characters such as different polarity or charge. PLTX-group toxins, with 2680 molecular weight (MW), have intermediate molecular weight; therefore the strategy envisaged here was to apply two separation mechanisms: size exclusion and chemical interactions.

##### 2.3.2.1. Pre-Purification of *Ostreopsis* cf. *ovata* Extract by Chemical Interaction Separation

Liquid-liquid extraction aimed to transfer undesirable molecules according to their polarity, from an extract to a non-miscible solvent, in order to purify and concentrate compounds. Hexane is often chosen to eliminate non-polar compounds; dichloromethane (DCM) is principally used to eliminate intermediate low polar compounds. Partition between *Ostreopsis* extract and different solvents has been reported by several authors. These extraction methods were generally not used alone but in combination with others, such as solid phase extraction [54,55] or flash chromatography [32]. However, efficiencies of these methods were not reported. Ovatoxins are not soluble in non-polar solvents such as chloroform, ether and acetone and sparingly soluble in methanol and ethanol, but soluble in pyridine, dimethylsulfoxide and water [1].

Initial trials examined partitioning of aqueous methanol against hexane or DCM. These experiments were also carried out in parallel after addition of acetic acid to enhance protonation and dissolution in the aqueous methanol layer. Mass balance of each organic phase showed that compounds transferred from the extract to the organic phases represented less than 2%, the vast majority of ovatoxins remaining in the aqueous methanol phase, together with the majority of compounds produced by *Ostreopsis* cf. *ovata*. This demonstrates that this first purification method is not suitable. Therefore, other purification methods were envisaged, using size exclusion methods.

##### 2.3.2.2. Pre-Purification of *Ostreopsis* cf. *ovata* Extract by Size Exclusion Separation

*Ostreopsis* cf. *ovata* extracts may contain high molecular weight compounds such as proteins or polysaccharides. First experiments aimed at eliminating these large compounds from *Ostreopsis* extracts by filtration through membranes possessing different cutoffs: 100,000 Da, 10,000 Da and 5000 Da. With molecular weight around 2680 Da, PLTX-group toxins should pass through all membranes. Crude extracts and filtrates were analyzed by LC-MS and compared. Preliminary results indicated that filtration through 100,000 and 10,000 Da membranes allowed the collection in the filtrate of the totality of toxins, however, this fraction also contained a lot of undesirable molecules. In contrast, 5000 Da cutoff membranes allowed for elimination of a large amount of undesirable compounds, but with only 56% of ovatoxins recovery in the filtrate. The capacity of palytoxin to form dimers, in aqueous solutions, could explain why a part of the PLTX-group toxins could not easily pass through the 5000 Da cutoff membrane [56]. Therefore, size exclusion chromatography appeared to be a more appropriate solution to purify ovatoxins.

**Figure 7:**
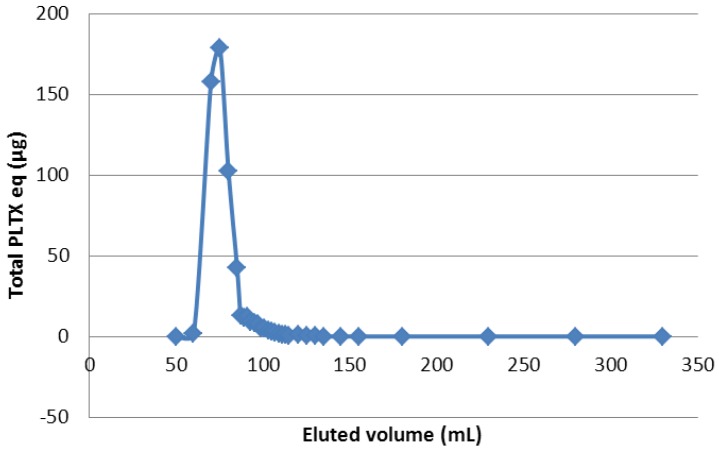
LC-MS/MS monitoring of p-PLTX and OVTX expressed as PLTX equivalent eluted through Sephadex LH-20.

The chromatographic sorbent Sephadex LH-20 is a stationary phase composed of highly cross-linked hydroxypropylated dextran on which separations can be obtained mainly on the basis of molecular size. Exclusion limit is around MW 5000 Da. With molecular weight of around 2680 Da, PLTX-group toxins would be partially excluded from this stationary phase. Depending on the solvent chosen, this medium can also separate sample components by partition between the stationary and the mobile phase. Two solvents, used as mobile phase, were investigated taking into account the solubility of the PLTX-group toxins: methanol and 50% aqueous methanol. The analysis of the collected fractions showed that the retention volumes for the toxins were the same, whatever the eluent, however, with 100% MeOH, the flow rate of eluent was higher, thus reducing elution time. PLTX-group toxin concentrations in the eluted fractions were monitored. Ovatoxins were obtained in fractions between exclusion and permeation volumes (Figure 7).

In order to determine the clean-up efficiency, fractions were gathered to obtain finally three parts; the first one corresponded to the exclusion volume of the column, before elution of toxins; the second one, the assembled fractions of the PLTX-group toxins and corresponded to the fraction of interest; and then, the third part corresponded to molecules eluting in the permeation volume of the column. Toxin quantitation of the fraction containing the toxins, by LC-MS/MS, showed that the majority of toxins was recovered, with *ca.* 85% yield.

The fraction of interest represented only 7% of crude extract mass (Table 3), 93% of undesirable compounds being contained in the other fractions. Among the extracts pre-purified with LH20, only one mass balance was carried out. Indeed, after total evaporation, re-dissolution of toxins was very difficult and only one third of the expected PLTX-group toxin was recovered. It was supposed that adsorption of toxins on the inner surface of the containers could occur. Therefore, for the following experimentations, purified extracts were concentrated to minimal volumes but not totally evaporated.

**Table 3:** Batch summary table for purification of PLTX-group toxin after elution through LH20.

Step	Weight (mg)	Toxin Quantity (mg)	Toxin Percentage (%)
**Cell Extract**	770	3.10	0.4
**LH20 (Fraction of Interest)**	54	2.76	5.1

Most of the extraction and purification processes found in literature for *Ostreopsis* extracts follow a general procedure with slight modifications. Generally, toxins were extracted with an aqueous-methanol mixture under sonication, sometimes with prior acidification [57,58]. The purification steps described include partition with hexane, dichloromethane and/or butanol [10,54], Solid Phase Extraction (SPE) with the stationary phase composed of C18 or ion exchange [5,59] or flash chromatography. Sometimes, several methods were combined [54]. Hwang (2013) used LH-20 to purify *Ostreopsis* extract, after partition and flash chromatography, the purification protocol being followed by preparative chromatography on a C18 column [55]. Little is known concerning efficiencies and yield obtained with these methods. Advantages of LH-20 were that it allowed the purification of large amounts of extract at once, with sufficient yield and a good repeatability. Additionally, it could be reused several times over long periods, unlike SPE cartridges or flash chromatography which need extensive regeneration before reuse. The possibility of automation, especially for flow rate or collection of fractions, could improve repeatability and make identification easier of the fractions of interest.

LH-20 chromatography provided elimination of a high quantity of undesirable compounds (93%) with good ovatoxin yield (89%) in the fraction of interest. However, in spite of the increase of toxin purity in this fraction (by 13-fold) toxin purification was not totally complete, so additional purification steps remained necessary. The selection of the stationary phase column for preparative chromatography is currently in progress.

## 3. Experimental Section

### 3.1. Sampling Site and Collection of Marine Organisms

During July 2011 and 2012, samples were collected by snorkeling at Villefranche-sur-Mer (South East of France), site called “Rochambeau” (43°41′34.83″ N and 7°18′31.66″ E). This site was characterized by a rocky bottom and sheltered depth, and regularly summer bloom of *Ostreopsis* cf. *ovata* had occurred in this area since 2006.

#### 3.1.1. Cell Abundance

To estimate the abundances of *Ostreopsis* cf. *ovata*, planktonic cells in the water column and epiphytic cells from macroalgae were collected and counted with the same protocol as Cohu *et al.*, 2013 [40]. Epiphytic cells were found on macoalgaes *Stypaucolon scoparium* and *Corallina elongata*.

For additional samplings, in order to evaluate toxin content of epiphytic *Ostreopsis* cf. *ovata vs.* depth, macroalgae *Padina pavonica* were sampled at 0.5, 1, 3 and 5 m. After isolation of micro and macroalgae [40], the suspension of toxic cells were separated into two parts: (i) one part for filtration under low pressure through a nylon net, filtered (hole width 20 μm) to separate cell pellets from seawater. The nylon net was then carefully closed to avoid any loss of cells and stored at −20 °C before extraction. After defrosting, cells were scrubbed in enough MeOH/H_2_O (1/1; v/v), to collect all pellets. Toxins were extracted and analyzed by LC-MS/MS as described below; (ii) *Ostreopsis* cells from the second part were evaluated with an optical microscope, using calibrated squared chambers (1 mL; Sedgwick Rafter) and then reported as number of cells per gram of fresh weight of macroalgae (cells/g·FW) [40].

#### 3.1.2. Isolation and Maintenance of Cultures of *Ostreopsis* cf. *ovata*

*Ostreopsis* cf. *ovata* cells were isolated by the capillary pipette method from Mediterranean water from near the seaweeds *Stypocaulon* sp. and *Acetabularia* sp. After initial growth in microplates, the cells were cultured in flasks at 22 °C under 16L:8D cycle (420 μmol·m^−2^·s^−1^). Culture conditions were previously optimized [27] and were established in filtered natural seawater, at salinity of 35, adding nutrients at L1 concentration and soil extract [60]. Cultures were uni-algal and clonal, but not axenic.

All cells of *Ostreopsis* cf. *ovata* cultured in the laboratory were isolated from around the Mediterranean area and were cultivated in a batch culture. A total of five different strains from France were cultivated in parallel; strains that were collected at Morgiret beach, near Marseilles in 2008 (IFR-OST-01M) and in 2009 (IFR-OST-02M) and at Villefranche-sur-Mer bay in 2008 (IFR-OST-01V), 2009 (IFR-OST-02V) and 2011 (IFR-OST-03V).

#### 3.1.3. Hemolytic Assay

Hemolytic assays were conducted following a method based on a combination of those described by Bignami (1993), Riobo *et al.* (2008) Aligizaki *et al.* (2008) [61,62,63]. Sheep erythrocytes were provided by Charles River Laboratories, L’Arbresle, France. Erythrocytes were separated from plasma by centrifugation (800 rpm, 5 min) and washed three times with 20 mL Dulbecco’s phosphate buffer saline (D-PBS, Sigma-Aldrich, St Quentin Fallavier, France). D-PBS contained 1 mM of calcium chloride and 0.5 mM of boric acid H_3_BO_3_. pHs were controlled between 7.0 and 7.2.

Two blood solutions were prepared: The first was pre-incubated 1 hour at 37 °C with ouabain (500 μM in D-PBS) a known antagonist of palytoxin, the other one was ouabain-free, both containing 0.5% (v/v) of sheep erythrocytes. The number of erythrocytes was checked as a quality control; the average of three determinations was 5.4 × 10^7^ cells. A 50 μL aliquot of samples or calibration solutions (Palytoxin Standard, Wako Chemicals GmbH, Neuss, Germany) prepared in triplicate was added to 950 mL of each red blood suspension and incubated 4 h at 37 °C. After the incubation, samples were centrifuged at 800 rpm over 8 min, 90 μL of the hemoglobin released supernatants were transferred in 96-well plates and the absorbance (A) was measured at 415 nm on a microplate reader (TECAN Infinite 200, Lyon, France).

Palytoxin standard solutions were prepared in triplicate by successive dilution between 500 and 0.06 ng·mL^−1^ to generate a sigmoid calibration curve. Marine organism samples were diluted 40 fold in D-PBS to avoid matrix effects. They were prepared in triplicate. Maximal lysis (A_max_) were determined by adding saponin (1%) to the blood preparation instead of the sample. Minimal lysis (A_min_) were determined adding D-PBS to the blood preparation instead of the sample.

%Lysis = (A − A*_min_*)/(A*_max_* − A*_min_*) × 100

### 3.2. Phylogeny

#### 3.2.1. DNA Amplification and Sequencing

Approximately 15 mL of exponentially growing cultures IFR-OST03V and IFR-OST01MO were harvested by centrifugation (4300× *g*, 10 min). DNA of pelleted cells was extracted using the CTAB (*N*-cetyl-*N*,*N*,*N*-trimethylammoniumbromide) method [64]. Since the ITS regions (ITS1 and ITS2) and 28S rDNA have been shown to be efficient to discriminate species of *Ostreopsis* [50], they were amplified by using oligonucleotide primers ITS-FW (5′-GTAGGTGAACCTGCGGAAGG-3′), and D3B (5′-TCGGAGGGAACCAGCTACTA-3′). Genomic DNA was amplified in 25 μL PCR reaction containing 1 μL of extracted DNA, 6.5 μL of ultrapure water, 2.5 μL of each primer (10 μM) and 12.5 μL of PCR Master Mix 1X (Promega, Madison, WI, USA) which includes Taq polymerase, dNTPs, MgCl_2_ and reaction buffer. The polymerase chain reactions were performed in a Mastercycler Personal (Eppendorf, Hamburg, Germany) as follows: one initial denaturating step at 94 °C for 2 min, followed by 45 cycles each consisting of 94 °C for 30 s, 54 °C for 30 s, and 72 °C for 4 min, and a final elongation at 72 °C for 5 min. The PCR products were visualized on a 1% (w/v) agarose gel, excised, and purified with the Wizard SV Gel and PCR Clean-up system (Promega, Madison, WI, USA) according to the manufacturer’s recommendations. Then, they were sequenced directly using the ABI PRISM BigDye Terminator Cycle Sequencing Kit (Applied Biosystems, Carlsbad, CA, USA). Sequencing products were purified by exclusion chromatography using the Dye Terminator Removal Kit (Thermo Scientific, Illkirch, France) and the sequences were determined using an automated 3130 genetic analyzer (Applied Biosystems, Carlsbad, CA, USA).

#### 3.2.2. Sequences Alignment and Phylogenetic Analysis

Molecular phylogeny was inferred from both a matrix of ITS1-5.8S-ITS2 and a concatenated ITS1-5.8S-ITS2-partial LSU matrix (data not shown). To prepare the ITS-5.8S dataset, the sequences of the two studied strains were aligned with 73 sequences of *Ostreopsis* and 7 sequences of *Coolia* retrieved in Genbank, using MUSCLE software [65]. The alignment was refined by eye using the BioEdit version 7.0.0. [66,67]. Evolutionary models were examined with jModeltest version 0.1.1 [68] Bayesian Inference (BI) analysis was run using Mr Bayes version 3.1.2 [69]. Initial Bayesian analyses were run with a GTR model (nst = 6) with rates set to invgamma and nucleotide frequencies set to equal. Each analysis was performed using four Markov chains (MCMC), with one million cycles for each chain. Trees were saved to a file every 100 cycles and the first 2000 trees were discarded. Therefore, a majority-rule consensus tree was created from the remaining 8000 trees in order to examine the posterior probabilities (pp) of each clade. Neighbor-joining (NJ) analysis was performed using MEGA software version 5.05 [70], with Maximum Composite Likelihood method. Bootstrap analysis (1000 pseudoreplicates) was used to assess the relative robustness of branches [71].

### 3.3. Evaluation of Cell Densities in Culture Media and Toxin Profile of Ostreopsis Strains

To evaluate cell concentration and toxin profile of strains of *Ostreopsis* cf. *ovata*, two 10 mL sample were carried out (i) the first sample was treated with HCl to a final concentration of 4 mM to dissolved mucus aggregate and cells were counted in triplicate. Cell concentration (cell·mL^−1^) was assessed using a particle counter (Multisizer 3 Coulter counter, Beckman, Roissy Charles De Gaulle, France). Only cells between 20 and 60 μm were counted to make sure of determining *Ostreopsis* sp. only, and not the contaminations eventually present in the culture media. Specific growth rate (μ·day^−1^) was calculated using the following equation: 
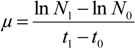
 where *N*_0_ and *N*_1_ are cell density values at time *t*_0_ and *t*_1_. (ii) the other one was used for extraction of toxin content without addition of HCl. Samples were harvested in triplicate, centrifuged at 3000× *g* over 15 min and the supernatant was discarded. Cell pellets were stored at −20 °C until extraction and analyzed by LC–MS/MS. (see Section 3.4.2. Extraction from cells). The rest of the biomass was collected and frozen to use for purification.

### 3.4. Extraction Procedure

#### 3.4.1. Chemicals

All organic solvents were obtained as HPLC grade solvents from JT Baker (Atlantic Labo, Bruges, France). Milli-Q water for mobile phase and extraction was supplied by a Milli-Q integral 3 system (Millipore, Saint-Quentin-Yvelines, France). Formic acid (Puriss quality), ammonium formate (Purity for MS), hexane and dichloromethane were from Sigma Aldrich (Saint Quentin Fallavier, France). Palytoxin for LC-MS/MS analysis was purchased from Wako Chemicals GmbH (Neuss, Germany), and dissolved in methanol-MilliQ water (1:1 v/v).

#### 3.4.2. Toxins Extraction from Cells

Toxins extraction from 10 mL of *Ostreopsis* cf. *ovata* culture or field epiphytic cells (pellets on nylon net) filtered were carried out according to the Amzil procedure [25] with slight modifications: (i) centrifugation at 3000× *g* at 4 °C for 15 min; (ii) recovery of the cell pellet in 1 mL of methanol/water (1/1;v/v); (iii) sonication with an ultrasonic probe twice, both over 40 min to make sure of the disruption of cell membranes, while cooling in an ice bath. Once all the cells were disrupted, the treated sample was centrifuged at 3000× *g* at 4 °C for 15 min. The supernatant was filtered through a 0.2 μm filter and the toxin profile determined by LC-MS/MS.

#### 3.4.3. Toxins Extraction from the Marine Organism

Each part of the marine organism was extracted according to the method developed by Amzil *et al.* [25]. The extraction was performed on 2 g of homogenates of tissue of shellfish (sea urchin, fishes and so on). The extraction was carried out successively 3 times with 3 mL of 90% MeOH, each extraction being followed by centrifugation at 3000× *g* for 15 min. Supernatants were combined and adjusted to 10 mL with 90% MeOH. An aliquot (300 μL) was filtered through a Nanosep MF 0.2 μm filter and analyzed by LC-MS/MS.

### 3.5. LC-MS/MS and LC-MS Analysis

LC-MS/MS experiments were performed using an LC system (UFLC XR, Shimadzu, Champs-sur-Marne, France) coupled to a hybrid triple quadrupole/ion-trap mass spectrometer (API 4000 Qtrap, ABSCIEX, Les Ulis, France) equipped with a turbospray interface. Toxins were separated on a 3 μm C18 Gemini column (150 × 2.0 mm, Phenomenex, Le Pecq, France), thermostated at 22 °C, with water (A) and 95% acetonitrile/water (B), both containing 2 mM ammonium formiate and 50 mM formic acid at 0.2 mL·min^−1^ flow rate. The gradient was raised from 20% to 100% B in 10 min and was held over 4 min before dropping down to the initial conditions.

Mass spectrum detection was carried out in multiple reactions monitoring (MRM) mode (positive ions). MRM experiments were established using source setting: curtain gas set at 30 psi, ion spray at 5000 V, a turbogas temperature of 300 °C, gas 1 and 2 set at 30 and 40 psi, respectively and an entrance potential of 10 V. To permit the best toxin identification, each toxin was quantified with three transitions as follows (Table 4).

**Table 4:** LC-MS/MS PLTX-group toxin transitions.

Toxins	[M + 2H]^2+^→Part A	[M + 2H − H_2_O]^2+^→Part A	[M + 3H − H_2_O]^3+^→Part A
**p-PLTX**	1340.3→327.3	1331.3→327.3	887.8→327.3
**OVTX-a**	1324.3→327.3	1315.3→327.3	877.2→327.3
**OVTX-b**	1346.3→371.2	1337.3→371.2	891.8→371.2
**OVTX-c**	1354.3→371.2	1345.3→371.2	897.2→371.2
**OVTX-d**	1332.3→327.3	1323.3→327.3	882.5→327.3
**OVTX-e**	1332.3→343.2	1323.3→343.2	882.5→343.2
**OVTX-f**	1338.3→327.3	1329.3→327.3	886.5→327.3

The collision energy was applied at 47 eV for bi-charged ions [M + 2H]^2+^, [M + 2H − H_2_O]^2+^ and at 31 eV for the tri-charged ion [M + 3H − 2H_2_O]^3+^ to give the characteristic product ion at *m/z* 327, 343 or 371 (Part A) [M + H − B moiety − H_2_O]^+^ (see section above, palytoxin structure). Declustering potential (DP) was set at 56 V for all transitions and cells exit potential (CXP) were at 20 V and 18 V for bi-charged ions and tri-charged ions, respectively. Transitions summarized in Table 4 were monitored with a dwell time of 25 ms for each transition. Because only the palytoxin standard was available, quantitative determination of putative-palytoxin, ovatoxin-a, b, c, d/e and f, in extracts was carried out assuming that their molar responses were similar to palytoxin, at concentrations of 50, 100, 500, 1000 ng·mL^−1^ and 2, 4, 8 10 μg·mL^−1^.

Full scan analyses were carried out using LC-MS experiments on an Agilent 1160 LC/MS (Agilent, Les Ulis, France) including solvent reservoir, online degasser, quaternary pump and thermostated autosampler. The MS analysis worked in positive mode, with mass range set at *m/z* 300–1450. The conditions of API-ES source were as follows; drying gas (N_2_); flow rate, 12 mL·min^−1^; drying gas temperature, 325 °C; nebulizer, 50 psig; capillary voltage, 4800 V; fragmentor 165 V. All the acquisition and analysis data were controlled by an Agilent LC/MSD ChemStation (Agilent, Les Ulis, France). Turning mix (G 2421-60001) was used for lock mass calibration in our assay.

Separation was carried out on a Kinetex 2.6 μm (100 × 4.6 mm) column (Phenomenex, Le Pecq, France). Linear gradient elution was accomplished in 20 min with water (eluent A) and 95% acetonitrile/water (eluent B) both containing 0.2% of acetic acid, at 0.7 mL·min^−1^ flow rate. The gradient was as follow: 0–20 min from 20% to 40% B, 20–21 min from 40% to 100% B, 21–28 min 100% B, 28–29 min from 100% to 20% B, and re-equilibration with 20% B. 

### 3.6. Extraction and Pre-Purification of Ovatoxins

#### 3.6.1. Evaluation of PLTX-Group Toxins Released in Culture Media

First experiments aimed to determine if an additional extraction method from media was necessary. Culture media of 25 days aged *Ostreopsis* cf. *ovata* culture was separated from the pellets by gravity filtration. After homogenization, media was separated into two equal parts; (a) the first part was extracted three times with an equal volume of butanol (BuOH). BuOH layers were combined and concentrated by a rotary evaporator. The sample was analyzed by LC-MS/MS; (b) the second part was extracted by passing through Diaion HP20 resin (Sigma-Aldrich, St Quentin Fallavier, France). 1 g of resin was first activated with MeOH over two hours, then transferred to a SPE column (6 mL) and conditioned with MilliQ water. The sample was loaded on resin. Each cartridge was first washed with MilliQ water (2 mL) and then eluted with 3 × 5 mL of MeOH. Samples were concentrated to 0.5 mL, filtered through a Nanosep MF 0.2 μm filter and analyzed by LC-MS/MS.

#### 3.6.2. Pre-Purification of *Ostreopsis* cf. *ovata* Extract by Chemical Properties

Twenty mL of *Ostreopsis* cf. *ovata* extract was prepared from 5 g of algal paste by a similar procedure as described in 4.4.2. After homogenization, the extract was separated into two equal parts. The first part was treated with 0.2% acid acetic in order to enhance toxin protonation. The other part was acid free. Both extracts were extracted in the same way: (a) 4.5 mL of the first and the second extract were partitioned three times with equal volumes of either hexane or dichloromethane, then the organic phases were combined. All phases (organic and aqueous phases) were evaporated. The residues were weighted, then dissolved in 5 mL of aqueous MeOH (1/1, v/v) and quantified by LC-MS/MS and analyzed by LC-MS. Mass balances were calculated as follow:

*m_fn_*/*m_ce_* × 100

where *m_fn_* is the weight of *n* evaporated fraction; *m_ce_* is the weight of the evaporated crude extract. Results are expressed as percentage of yield.

#### 3.6.3. Pre-Purification of *Ostreopsis* cf. *ovata* Extract by Chemical Separation

##### 3.6.3.1. Experiment 1: Filtration on Membranes

A volume of 5 mL of *Ostreopsis* cf. *ovata* extract was prepared from 1 g of algal paste. After homogenization, 12 × 300 μL of extract were filtered with four different membranes (vivaspin, Sartorius) to obtain triplicates for each membrane: (a) 100,000 Da cutoff; (b) 10,000 Da cutoff; (c) 5000 Da cutoff; (d) 0.2 μm filtration membrane usually used before LC-MS/MS analysis. Each filter was centrifuged at 10,000 rpm over 1 h. Filtrates were quantified by LC/MS-MS and analyzed by LC-MS.

##### 3.6.3.2. Experiment 2: LH20 Chromatography

A volume of 100 mL of *Ostreopsis* cf. *ovata* extract was prepared from 20 g of algal paste. This extract was pre-purified with Sephadex LH20 column. Prior to use, 10 g of LH20 sorbent was conditioned with MeOH over one night, then packed in a glass column (80 × 2 cm) and finally rinsed with MeOH. Concentrated *Ostreopsis* cf. *ovata* extract was deposited on top of the column; compounds were then eluted with MeOH. Eluting fractions were collected, filtered through a Nanosep MF 0.2 μm filter, quantified by LC-MS/MS and analyzed by LC-MS.

Fractions were gathered to obtain three fractions (a) the first one contained compounds eluting before elution of PLTX-group toxins; (b) the second containing all PLTX-group toxins; (c) the third contained compounds eluting after PLTX-group toxins. Fractions and crude extract were analyzed by LC/MS-MS, and then totally evaporated and weighed. Mass balance was calculated as followed:

*m_fn_*/*m_ce_* × 100

where *m_fn_* is the weight of *n* fraction evaporated; *m_ce_* is weight of crude extract evaporated. Results are expressed as percentage of yield.

## 4. Conclusions

This study clarifies some aspects of the eco-physiology of *Ostreopsis*, in particular concerning cellular growth and toxin production. *Ostreopsis* cf. *ovata* regularly blooms in the French Mediterranean coastal waters, sometimes at very high cell concentrations. Toxin production of ovatoxins was observed down to 3 m depth; indeed, this report is the first indication that the contents of ovatoxins decrease with increasing depth. Risks due to *Ostreopsis* bloom are real, but due to lack of toxin standards, the danger is not well characterized, in particular for food intoxication. Here, we propose a pre-purification method for ovatoxins from the biomass of cultured *Ostreopsis* cf. *ovata*, using a chromatographic separation with a Sephadex-LH20 phase. This step allowed an elimination of 93% of undesirable compounds and permitted a recovery of 85% ovatoxins. Further investigations are in progress to finalize the isolation of ovatoxins, in order to allow further in-depth toxicity studies and finally to help sanitary authorities to make decisions for the efficient protection of bathers and seafood consumers.

## Supplementary Files

Supplementary File 1 PDF-Document (PDF, 48 KB)
