# Supplementary material for: Complex Toxin Profile of French Mediterranean Ostreopsis cf. ovata Strains, Seafood Accumulation and Ovatoxins Prepurification
Source: Mar Drugs. 2014 May 13;12(5):2851–76. doi: 10.3390/md12052851 (PMC4052321; doi:10.3390/md12052851)
Supplement: Supplementary File 1 — PDF-Document (PDF, 48 KB) [file marinedrugs-12-02851-s001.pdf]

Supplementary Information

Table S1. Percentage of toxins according to the depth.

Depth (m)	p-PLTX (%)	OVTX-a (%)	OVTX-b (%)	OVTX-c (%)	OVTX-d (%)	OVTX-e (%)	OVTX-f (%)
**0,5**	13	48	13	8	5	12	0
**1**	17	37	17	11	2	16	0
**3**	15	38	14	12	4	16	0

Table S2. Summary of total PLTX-group toxins concentrations found on different contaminated marine organisms. Sea urchins diameters without spines and sea-breams total length were indicated in brackets.

**Marine Organisms Number of Organisms Sampled**	**Sea Urchins (Paracentrotus Lividus) *n* = 10 (Diameter without Spines from 4 and 5 cm)**		**Sea Breams (Sarpa Salpa) *n* = 21 (Total Length from 7.1 and 9.8 cm)**	
**Number of Organisms Contaminated**	9		6	
**Total PLTX-eq in whole Flesh of Contaminated Organism (µg/kg) (Size of Organisms cm)**	231	(4.5)	54	(7.8)
	213	(4)	85	(7.9)
	309	(4)	33	(7.7)
	205	(4)	152	(7.1)
	103	(5)	19	(9.8)
	191	(4.5)	54	(9.2)
	423	(4.5)		
	215	(4.5)		
	121	(4)		

Figure S1. Nonlinear regression of *Ostreopsis* cf. *ovata* in function of depth.

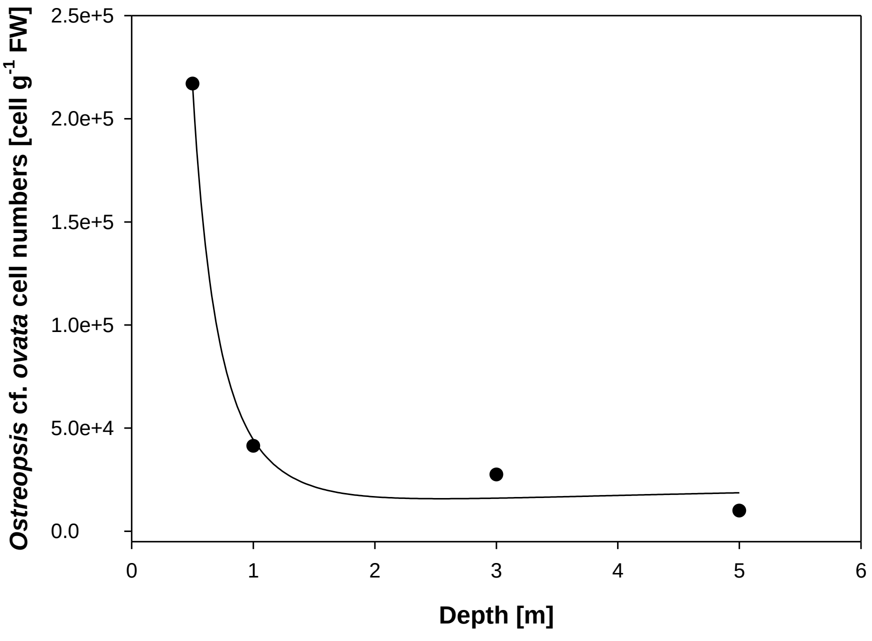

Figure S1. *Cont.*

Nonlinear Regression

Data Source: Data 1 in Notebook1
Equation: Polynomial, Inverse Second Order
f = y0+(a/x)+(b/x^2)

R	Rsqr	Adj Rsqr	Standard Error of Estimate
0.9961	0.9923	0.9768	14672.6894

	Coefficient	Std. Error	t	P
y0	27751.8472	19006.5881	1.4601	0.3823
a	-60949.3960	50731.9512	-1.2014	0.4419
b	77646.7708	22434.7535	3.4610	0.1791

Analysis of Variance:

	DF	SS	MS	
Regression	3	49428568586.8602		16476189528.9534
Residual	1	215287813.1398	215287813.1398	
Total	4	49643856400.0000		12410964100.0000

Corrected for the mean of the observations:

	DF	SS	MS	F	P		
Regression	2	27580772486.8602		13790386243.4301		64.0556	0.0880
Residual	1	215287813.1398	215287813.1398				
Total	3	27796060300.0000		9265353433.3333			

Statistical Tests:

Normality Test (Shapiro-Wilk) Passed (P = 0.8162)

W Statistic= 0.9659 Significance Level = 0.0500

© 2014 by the authors; licensee MDPI, Basel, Switzerland. This article is an open access article distributed under the terms and conditions of the Creative Commons Attribution license (http://creativecommons.org/licenses/by/3.0/).
